# Group problem management plus (gPM+) in the treatment of common mental disorders in Syrian refugees in a Jordanian camp: study protocol for a randomized controlled trial

**DOI:** 10.1186/s12889-020-08463-5

**Published:** 2020-03-26

**Authors:** Aemal Akhtar, Luana Giardinelli, Ahmad Bawaneh, Manar Awwad, Hadeel Naser, Claire Whitney, Mark J. D. Jordans, Marit Sijbrandij, Richard A. Bryant, on behalf of the STRENGTHS Consortium

**Affiliations:** 1 School of Psychology, University of New South Wales, Sydney, NSW 2052 Australia; 2 Clinical, Neuro and Developmental Psychology, VU University, Amsterdam, The Netherlands; 3 Jordan Country Office, International Medical Corps, Amman, Jordan; 4 International Medical Corps, Beirut, Lebanon; 5 Research and Development Department, War Child Holland, Amsterdam, The Netherlands; 6 Amsterdam Institute of Social Science Research, University of Amsterdam, Amsterdam, The Netherlands

**Keywords:** Refugees, Mental health, Behavioural intervention, Controlled trial, Psychosocial intervention

## Abstract

**Background:**

Accessing quality mental health care poses significant challenges for persons affected by adversity, especially in low- and middle-income countries where resources are scarce. To mitigate this, the World Health Organization has developed group problem management plus (gPM+), a low-intensity psychological intervention for adults experiencing psychological distress. gPM+ is a group-based intervention consisting of five-sessions, and can be delivered by non-specialist providers. This paper outlines the study protocol for a trial of gPM+ in Jordan.

**Methods:**

We will conduct a single-blind, two-arm, randomized controlled trial in a Syrian refugee camp in Jordan. We aim to enrol 480 adults into the trial. Participants will be eligible for the trial if they screen positive for levels of psychological distress. Following screening, those eligible will be randomly assigned to receive the gPM+ intervention or enhanced treatment as usual. The primary outcome is reduction in levels of psychological distress at 3-months post-treatment. Secondary outcomes include anxiety, depression, prodromal psychotic symptoms, posttraumatic stress disorder, prolonged grief, daily functioning, economic effectiveness, and change in parenting behaviour. Secondary outcomes also include the reduction in psychological distress of the participant’s child.

**Discussion:**

The trial aims to deliver a template for affordable and scalable psychosocial interventions that can readily be implemented in refugee settings, and that can benefit both the participant and their child.

**Trial registration:**

Australian New Zealand Clinical Trials Registry, ACTRN12619001386123. Registered prospectively on 10/10/2019.

## Background

Globally, there are currently more than 70 million displaced people, with over 25 million refugees [1]. Refugees are exposed to many distressing and potentially traumatic events, including war, sexual violence, torture, dangers faced while fleeing their homeland, and risks associated with being confined in camps or detention centers. It is not surprising that refugees have been shown to experience elevated rates of mental health problems, including depression, anxiety, suicide risk, posttraumatic stress disorder (PTSD), and somatic conditions [2–4]. The largest population of refugees are Syrians who have fled from the 2011 Syrian civil war. UNHCR projections indicate that 15–20% of Syrians will experience mental health conditions in the aftermath of the adversity they have faced, thus highlighting a need for effective interventions for conflict-affected populations [5, 6]. Evidence-based mental health programs have been shown to be efficacious in refugee populations [7–9], with meta-analysis indicating they have a moderate effect [10].

Despite the potential of these interventions, there are often obstacles to implementation of these programs in countries hosting refugees. These programs have generally not been scaled up in many settings of humanitarian crises, or in those hosting large numbers of refugees, because these interventions: (a) tend to only target a single diagnostic outcome [11], (b) are generally resource intensive [12], and (c) require professionals or extensively trained and supervised lay therapists [13]. Like most humanitarian settings, countries hosting a vast number of refugees are often not adequately resourced to provide the services necessary to address the mental health needs [14]. In response, there has been a move towards ‘task-shifting’ programs that train non-specialists in simple mental health programs to increase the workforce capacity to implement mental health programs in low and middle income countries (LMIC) [15]. A recent meta-analysis of task-shifting programs indicated they yielded a moderate effect size in reducing psychological distress [16].

In line with its Mental Health Gap Action Programme, the World Health Organization (WHO) utilised a task-shifting approach to inform the development of an intervention that could be delivered in an affordable manner in under-resourced settings by trained lay providers. The transdiagnostic intervention aimed to effectively reduce common mental disorders [17]. This program, Problem Management Plus (PM+), comprises five sessions that teach participants strategies in problem-solving, behavioral activation, arousal reduction, and accessing social support [18]. This program has been subjected to multiple large-scale randomised controlled trials, and has been shown to be effective when delivered in individual [19, 20] and small-group formats [21]. Although PM+ has been shown to be effective across different populations (survivors of civil unrest and gender-based violence) [19, 20], it has yet to be shown to be effective in alleviating distress in refugees. Accordingly, one goal of the current trial is to assess how efficacious group-based PM+ is in reducing psychological distress in refugees. This study focused on refugees in a camp environment because this setting can involve specific stressors, including detention, lack of employment opportunities, and restriction to one’s social network (including internet access).

A second goal of the study is to index the extent to which PM+ administered to adult refugees may have beneficial effects on their children. Most of the world’s refugees are under 18 years of age, and they are at risk for elevated rates of psychological difficulties [1, 22]. This pattern is also observed in the context of Syrian refugees, of whom more than 50% are youth [23, 24]. One major factor that can impact a refugee child’s mental health is the psychological status of their parents or caregivers [25, 26]. Specifically, psychological difficulties can adversely affect parenting behavior, which can in turn contribute to psychological impairment in children [17]. Accordingly, although PM+ is only administered to adult refugees, this study aimed to assess the extent to which PM+ administered to caregivers also improved the mental health of the participants’ child.

## Methods/design

### Aim and design

This protocol represents Version 1.0 of this trial (dated 20th January, 2020); any alterations to the trial protocol will be updated on the ANZCTR registry. We will conduct a two-arm, single-blind randomised controlled trial (RCT) comparing Group Problem Management Plus (gPM+) to enhanced treatment as usual (ETAU) in 480 study participants. See Table 1 for an overview of the Standard Protocol Items: Recommendations for Intervention Trials (SPIRIT) [27]. The primary aim of this study is to evaluate the effectiveness of a locally adapted version of gPM+ on symptoms of psychological distress in male and female Syrian refugee adults in Jordan. The secondary aims are to assess (1) the extent to which gPM+ improves the mental health of the participants’ child, and (2) to assess the effectiveness of gPM+ on a range of other measures of adult mental health. Participants will be assessed at baseline, post-intervention, and three and 12 month follow-ups. The primary outcome time point is the 3-month assessment.

**Table 1:**
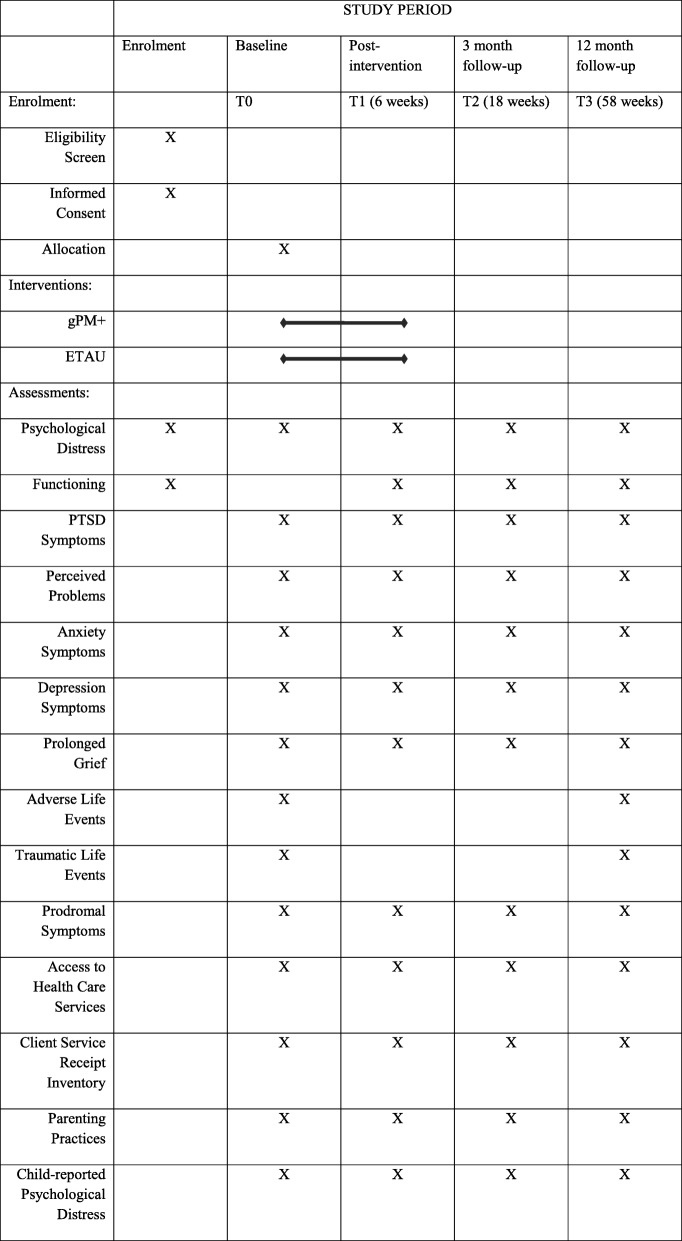
Standard protocol items recommendations for intervention trials (SPIRIT): Schedule of enrolment, interventions, and assessments for STRENGTHS trial

### Setting

Jordan is one of the countries most affected by the Syria crisis, hosting the second highest share of refugees per capita. In total, 654,681 Syrian refugees are registered in Jordan with the government estimating that more than 1.4 million Syrians are currently residing in Jordan. The study will be conducted in Azraq Refugee Camp, Jordan. Azraq camp first opened in 2014, and currently hosts 36,010 Syrian refugees across four villages, 60% of which are children. The study will be overseen by staff at International Medical Corps (IMC) Jordan.

### Participants

Participant inclusion criteria are: a) adults aged 18 years or older, b) scores above 16 on the WHO Disability Assessment Schedule 2.0 (WHODAS 2.0) [28] screener, c) scores above 15 on the Kessler Psychological Distress Scale (K10) [29], and d) have a child between the ages 10–16 years,. Exclusion criteria are: a) significant cognitive or neurological impairment, b) acute medical conditions, c) severe mental disorders (e.g. psychotic or substance-abuse disorders), and d) acute risk of suicide.

### Informed consent and assent

Informed consent entails a two-step procedure: 1) informed consent to conduct the screening and 2) informed consent for taking part in the gPM+ trial. The latter is only required for participants meeting the inclusion criteria following screening. For each step, participating respondents will be asked to complete a written consent form. For participants who are illiterate, witnessed oral consent will be collected, in line with recommendations from the WHO [30]. Following screening, participants will receive feedback on their results. For those who screen positive, the assessor will schedule a follow up visit and invite them to complete the pre-assessment and participate in the trial.

For participants who consent to taking part in the trial, we will ask if we can obtain assent from one child between the ages of 10 and 16 years. If permission is granted, we will then approach the child for assent. Children’s assent is not required for the participation in the study.

### Procedure

Assessors hired and trained by UNSW/International Medical Corps (IMC) will identify participants through door-to-door screening in the camp. The assessors will be randomly assigned pre-specified areas within the camps villages. At each caravan, assessors will provide a short explanation of the study and purposes of screening, then ask whether there is an adult who would be willing to participate. After consent has been provided, the assessor will record socio-demographic data and ask the participant to complete the WHODAS 2.0, K10, and a brief prolonged grief screener (PG-S). The assessor will additionally screen for imminent risk of suicide and neurological impairment.

All screenings will be conducted face-to-face in the participant’s home unless the assessor deems the location unsuitable due to privacy, in which case they will conduct the screening in pre-determined secure locations. If participants are not selected because they score below the cut-offs for the WHODAS 2.0 or the K10, they will be provided feedback on their test outcomes and reasons why they are not eligible for the study will be explained to them. If participants meet any of the exclusion criteria, assessors will refer cases to their clinical supervisor who will subsequently refer them to specialised services in accordance to interagency standard operating procedures. If participants screen positively and are not excluded due to neurological impairment or suicide ideation, assessors will schedule a follow up appointment to obtain consent to participate in the trail and administer the pre-assessment. Pre-assessment consists of the following instruments: Hopkins Symptom Checklist (HSCL-25) [31], Traumatic Events Checklist (TEC) [32], Post-Migration Living Difficulties (PMLD) [33], the PTSD Checklist for DSM-5 (PCL-5) [34], Psychological Outcome Profiles (PSYCHLOPS) [35], Access to Health Care Services (AHCS), Client-Service Receipt Inventory (CSRI) [36], the Prodromal Questionnaire – Brief (PQ-B) [37], Prolonged Grief-13 scale (PG-13) [38], and the Alabama Parenting Questionnaire (APQ) [39]. If the participant’s child provides assent, we will additionally ask them to complete the child-reported version of the Pediatric Symptoms Checklist-35 [40].

The post-intervention assessment is scheduled 7 weeks after the pre-intervention assessment (i.e., 1 week after the 5th PM+ session), and the follow-up assessment is scheduled at 3 months after the post-intervention assessment (i.e. 20 weeks after inclusion, in line with the timing of the follow-up assessment for the PM+ participants). Table 1 presents an overview of measures that are administered at each of the assessments.

The assessment packages will be uploaded onto tablets and participants will be asked to complete them on the tablets; assessors fluent in Arabic will be present to clarify questions. The assessors will deliver select instruments in an interview format because of the sensitivity or difficulty of questions (e.g. thoughts of suicide; health service use).

Prior to taking part in the study, assessors will receive a four-day training regarding psychological first aid, research ethics, administration techniques for each questionnaire, data collection, general interviewing techniques, and an introduction to common mental disorders. Ongoing monitoring of assessors’ competency will be conducted through regular supervision by the trial manager. The assessors will be blind to the allocation status of the participants.

### Randomisation

Randomisation will occur following the completion of the pre-assessment. The randomization sequence will be generated by an independent research assistant located off-site (University of New South Wales) who is not involved in any other aspect of the study. Randomisation will be performed using computerized software on a 1:1 basis. A research assistant employed by IMC who is not involved with any other aspect of the study will allocate personnel to the group they are randomised into and invite them to the first session of gPM+.

### Interventions

#### The group problem management plus (gPM+) programme

The WHO PM+ programme involves a set of a brief psychological interventions that seek to ameliorate symptoms of common mental health problems (e.g. depression, anxiety). The intervention protocol was written by a consultant at the University of New South Wales, Australia [18, 41].

The PM+ manual then underwent a comprehensive cultural and contextual adaptation process to ensure it would be appropriate for Syrian refugees, and then further refined to the local sociocultural context of the lives of Syrians residing in Jordan. The manual was translated into Arabic, after which the translation was reviewed in cultural adaptation workshops.

PM+ integrates problem-solving and behavioural treatment techniques that demonstrate amenability to low-intensity delivery and are evidence-based [42–45]. gPM+ is delivered over 5 weekly sessions of 120 min duration. Clients are systematically taught four strategies, including stress management, problem management, behavioural activation, and skills to strengthen social support. In the current RCT, the group PM+ format will be tested in anticipated group size of 8–10 participants. Groups are conducted separately for men and women.

Each session will be conducted by two gPM+ providers; a facilitator and a co-facilitator. The gPM+ providers will be non-specialists, who are recruited by IMC, Jordan. gPM + providers will hold a bachelor degree in a psychology or a field related to health (form 4 level of education or above) and have proficiency in Arabic. In addition, one local supervisor who works within the camp will be employed. The facilitators will receive 8 days of training in the delivery of the group PM+ intervention as well as basic counselling skills and group facilitation skills. Following training, the gPM+ providers will be required to complete two practice cycles, as a lead facilitator and as a co-facilitator, under close supervision.

Protocol adherence will be ensured by the supervisors and weekly group supervisions of the facilitators [46]. Supervisors will receive weekly supervision and on-the-job training in supervision skills by a PM+ master trainer to ensure treatment adherence and to provide additional supervisory support as needed.

To evaluate treatment fidelity, 10% of all PM+ sessions will be attended by the supervisor, using a checklist to ensure basic elements of the PM+ intervention have been followed as required. Facilitators will also complete self-evaluation forms and discuss challenges with the supervisor.

#### Enhanced treatment-as-usual (ETAU)

Participants who are randomised into the ETAU group will receive a home visit from IMC staff and will be provided with information on the organisations present in the camp where they can seek help for mental health concerns as well as a range of other activities pertaining to health, parenting, and vocational training. Those randomised into the ETAU arm will not be offered PM+ for the duration of this study.

We will track the types and amount of support participants receive during the time they are enrolled in the study through the CSRI. If during this treatment or during the study’s assessments participants in ETAU arm show severe psychiatric disorders or problems (e.g. psychosis or suicidality) that require immediate specialist treatment, they will be referred to IMC mental health clinics for further assessment and intervention. If risk of harm for themselves or others is determined, participants will be referred to the National Centre for Mental Health in Jordan.

#### Screening measures

The WHODAS 2.0 [28] is a generic assessment instrument assessing general functioning, health and disability. WHODAS covers six domains (cognition, mobility, self-care, getting along, life activities, and participation) and assesses difficulties people have due to their illness across these domains during the last 30 days. Difficulties are scored as none, mild, moderate, severe, or extreme. We will use the 12-item interviewer administered version and the recommended cut-off score of 17 will be used.

The Kessler Psychological Distress Scale (K10) is a questionnaire assessing general psychological distress [29]. It consists of 10 items indexing anxiety and depression symptoms that are experienced during the past 30 days. Responses are scored on a scale of 1 (none of the time) to 5 (all of the time) with scores ranging from 10 to 50. Higher scores are indicative of higher levels of psychological distress. A cut-off score of 16 will be used which has previously been shown to be indicative of moderate levels of distress [42, 43].

#### Primary outcomes

The primary outcome is levels of psychological distress as determined using the Hopkins Symptom Checklist (HSCL-25). The HSCL-25 consists of 25 questions across subscales of anxiety (10 items) and depression (15 items) related to the level of impairment or distress caused by symptoms. Responses are rated on a 4-point categorical scale (1 = *not at all*, 4 = *extremely*). Total scores are calculated by taking the average of the responses while depression and anxiety specific scores are calculated by taking the average of the related questions.

#### Secondary outcomes

Posttraumatic stress disorder (PTSD) symptoms will be measured using the PCL-5 [33], which is a 20-item checklist corresponding with the 20 DSM-5 PTSD symptoms. Items are rated on a 5-point scale (0 = *not at all*, 4 = *extremely*) scale and add up to a total severity score of 80. For the post-assessment, the PCL-5 will be adapted to ask for symptoms in the last week (rather than month) to enhance sensitivity to change.

PSYCHLOPS [35] assesses progress on problems for which the person seeks help. It consists of four questions that encompass three domains: problems (2 questions), functioning (1 question) and wellbeing (1 question). Participants are asked to give free text responses to the problem and function domains. Responses are scored on an ordinal six-point scale producing a maximum score of 20 (5 points per question. The PSYCHLOPS version administered at posttreatment and follow-up also includes an overall valuation question (determining self-rated outcome ranging from “much better” to “much worse”). PSYCHLOPS has been validated in primary care populations across several countries [44, 45].

Parenting behaviours will be assessed using the Alabama Parenting Questionnaire-42 (APQ-42). The APQ consists of 42 items measuring various disciplinary practices. Five constructs are measured: (1) involvement, (2) supervision and monitoring, (3) positive parenting, (4) consistent discipline, and (5) corporal punishment. Additional items not utilized in the five constructs assess other forms of disciplinary measures. All items are rated on a 5-point scale (1 = never, 5 = always). Construct scores are calculated by summing item scores.

Prolonged grief will be assessed using the PG-13 [38]. The PG-13 is a 13-item self-report measure that indexes the core symptoms of prolonged grief disorder (PGD). Each symptom is rated on a 5-point scale (1 = *not at all*, 5 = *overwhelmingly*). It is the most widely used measure of PGD, represents a unidimensional scale, and has been shown to index grief-related impairment. The PG-13 items converge with ICD-11 criteria for PGD.

Prodromal psychotic symptoms will be assessed using the Prodromal Questionnaire-16 (PQ-B16) [37]. The self-reported questionnaire consists of 16 true or false items; items that are endorsed have additional questions that ask about levels of distress experienced for the endorsed symptoms on a 4-point scale (0 = *no*, 3 = *severe*). Respondents who report true for six or more items are considered to be at risk for developing psychosis.

Psychological distress in the children of participants will be assessed using the youth-reported version of the Pediatric Symptoms Checklist [40]. It comprises 35 items rated on a 3-point scale (0 = *never*, 2 = *often*) and measures symptoms of internalizing, externalizing, and somatic symptoms. The total score is calculated by summing the scores of the individual items and ranges from 0 to 70.

#### Other measures

Previous exposure to traumatic events will be assessed using the TEC. The TEC contains 27 potential events that the participants may have witnessed during their displacement and subsequent time residing in the camp. The PMLD will be used to assess specific difficulties experienced by the Syrian refugees upon arriving to Jordan. The PMLD has 17 items which are rated on a 5-point scale (0 = was not a problem, 4 = a very serious problem). Access to health care services and the associated costs will be captured using the AHCS and CSRI questionnaires, both of which were adapted to the local context [36]. The AHCS asks the participant to identify whether they have had emotional or behavioural problems in the past and whether they have previously received any form of mental health care services. The CSRI will be used to index service utilization prior to and throughout the study and other health-care indicators to calculate the cost of care [46].

### Data management and analysis

A local Study Safety Committee that comprised three Jordanian health professionals will function to monitor any adverse events that occur during the trial. All adverse events will be reported by assessors or PM+ facilitators to this committee, who will ensure that appropriate action is taken by referral to local services. Serious adverse events will be entered in the Castor EDC trial monitoring software and reported to the Study Safety Committee and also to the STRENGTHS Central Committee on Research Involving Human Subjects within 7 days (in case of death or life-threatening situation) or 15 days (all other events). A Data Monitoring Committee is not formed because multiple trials of PM+ indicate that it is a safe program and will not cause harm. Accordingly, there is no likelihood that the trial will need to be halted prior to its completion.

A total number of 480 participants will be included in the trial. Based on previous studies of PM+, we aimed for a conservative effect size of 0.4 in the PM+ group at 3-months. Power calculations suggest a minimum sample of 133 per arm (power = 0.90, α=). 05, two-sided). Taking into account an expected 40% attrition at 3-month follow-up, based on a feasibility study, we aim to include a total of 240 participants per arm.

All data will be de-identified to protect personal information. Bryant and an independent statistical consultant will independently have access to the data and conduct analyses after the blind is broken after the 3-month follow-up. Hierarchical linear modeling (HLM) analysis will be conducted to assess differential change over time in HSCL-25 scores between groups. For each outcome, the effects of time of measurement, group, and the group-by-time interaction will be analyzed. HLM presumes intent-to-treat analyses as HLM allows the number of observations to vary between participants and effectively handles missing data. Time (linear and quadratic), treatment condition, and their interaction will be included in the models. Fixed effects parameters will be tested at 95%CI. The Level 1 model will represent within-patient change over time, and the Level 2 model will predict variation in within-patient change over time and encompass between-patient variables. Across all analyses, two-tailed tests will be reported with *p* < .05.

### Ethics

The project has been approved locally by the institutional review board at the King Hussein Cancer Centre in Amman, Jordan, and the University of New South Wales Human Research Ethics Committee.

## Discussion

In the context of the increasing number of refugees across the world, there is an ongoing need to evaluate scalable programs that can reduce the common mental disorders that are well-documented in refugees. The specific challenges of living in a refugee camp, in addition to the many adversities experienced by those who have fled war and persecution, highlights the need for programs that can assist people to psychologically cope in these settings. In recognition that most countries hosting refugees have limited resources, it is imperative that any psychosocial programs are affordable in the context of countries’ available resources. It is to this end that this project aims to extend current endeavors in task-shifting to evaluate the extent to which PM+ can reduce distress and improve functioning in Syrian refugees in this camp environment. For this reason these results will be published in in international journals, and disseminated in Arabic to local providers in Jordan. Moreover, there is potentially great opportunities to improve parenting behavior and children’s mental health by enhancing the psychological well-being of parents and caregivers.

## Data Availability

The data is being made available to the STRENGTHS Consortium initially in order to allow individual patient data meta-analyses of all PM+ trials being conducted within this consortium. Following this, data will be freely available on request.

## Abbreviations

AHCS: Access to health care services
APQ: Alabama parenting questionnaire
CSRI: Client-service receipt inventory
ETAU: Enhnaced treatment as usual
gPM +: Group problem management plus
HSCL-25: Hopkins symptom checklist
K10: Kessler psychological distress scale
LMIC: Low and middle income countries
PCL-5: PTSD Checklist for DSM-5
PQ-B: Prodromal Questionnaire – Brief
PG13: Prolonged grief-13
P CL-5: PTSD Checklist for DSM-5
PMLD: Post-Migration living difficulties
PSYCHLOPS: Psychological outcome profiles
HLM: Hierarchical linear modeling
TEC: Traumatic events checklist
UNHCR: United Nations High commissioner for refugees
WHO: World Health Organization
WHODAS: WHO disability assessment schedule 2.0
